# An improved ant colony optimization algorithm with fault tolerance for job scheduling in grid computing systems

**DOI:** 10.1371/journal.pone.0177567

**Published:** 2017-05-17

**Authors:** Hajara Idris, Absalom E. Ezugwu, Sahalu B. Junaidu, Aderemi O. Adewumi

**Affiliations:** 1 Department of Mathematics, Ahmadu Bello University Zaria, Nigeria; 2 Department of Computer Science, Federal University Lafia, Nasarawa State, Nigeria; 3 School of Mathematics, Statistics and Computer Science, University of Kwazulu-Natal, Westville Campus, Durban, South Africa; Southwest University, CHINA

## Abstract

The Grid scheduler, schedules user jobs on the best available resource in terms of resource characteristics by optimizing job execution time. Resource failure in Grid is no longer an exception but a regular occurring event as resources are increasingly being used by the scientific community to solve computationally intensive problems which typically run for days or even months. It is therefore absolutely essential that these long-running applications are able to tolerate failures and avoid re-computations from scratch after resource failure has occurred, to satisfy the user’s Quality of Service (QoS) requirement. Job Scheduling with Fault Tolerance in Grid Computing using Ant Colony Optimization is proposed to ensure that jobs are executed successfully even when resource failure has occurred. The technique employed in this paper, is the use of resource failure rate, as well as checkpoint-based roll back recovery strategy. Check-pointing aims at reducing the amount of work that is lost upon failure of the system by immediately saving the state of the system. A comparison of the proposed approach with an existing Ant Colony Optimization (ACO) algorithm is discussed. The experimental results of the implemented Fault Tolerance scheduling algorithm show that there is an improvement in the user’s QoS requirement over the existing ACO algorithm, which has no fault tolerance integrated in it. The performance evaluation of the two algorithms was measured in terms of the three main scheduling performance metrics: makespan, throughput and average turnaround time.

## Introduction

Grid emerges from solving computational problems which otherwise cannot be solved by an individual or stand-alone computing systems. This extremely high computing power is achieved by the optimal utilization of distributed heterogeneous resources which are lying idle. This has enabled scientists to broaden their simulations and experiments to take into account more parameters than ever before. Owning to the fact that high performance computing resources are expensive and hard to access, alternative choices are to use federated resources that could comprise computation, storage and network resources from multiple geographically distributed institutions [1]. As most systems are idle for significant periods of time, it should be possible to harness their idleness or unused resources and apply them towards projects in need of such resources. The Grid paradigm emerged, as a result of the resourceful contributions made by Foster, Carl Kesselman, and Steve Tuecke [2, 3]. Their work put together, led to the development of the Grid toolkit, that handled computation management, data movement, storage management and other infrastructure that could handle large Grid computations without restricting themselves to specific hardware and requirement [4].

Due to the dynamic nature of the Grid computing environment, more resources failures are likely to occur in the environment, which may affect the actual execution time required to execute already scheduled jobs and thereby degrading the performance of the system. Grid compute intensive applications or jobs as the case may be and often require much longer execution time in order to solve a single problem. The huge computing potential of Grid systems usually remain unexploited due to their susceptibility to failures, such as process failures, machine crashes, and network failures [5, 6, 7]. The failure of a resource running a user job has a huge setback on the Grid performance. Hence, in order to ensure high system availability, job site failure handling is inevitable. In Grid computing, incorporating fault tolerant algorithms in the course of job scheduling process is often advocated. It is in this light, that an extension of the work proposed in [8] is extended in this paper by incorporating a fault tolerant scheduling algorithm into the Swarm Intelligent Grid Job Scheduling Algorithm proposed by the author. The existing algorithms however, do not take into consideration Grid resource failure.

Fault tolerance is responsible for handling the reliability and availability of distributed systems [9]. Fault tolerance is a capability developed in the system so that it could perform its function correctly even in the presence of resource failure. It is developed to detect immediately the occurrence of faults and recover the executable task without participation of any external agents, thereby, making the system more dependable. In fault tolerance, according to Garg and Kumar [10], failure is encountered when a system drifts away from its normal behavior. The cause of a failure is called error, which also ultimately depicts some sort of fault or defect in that system. That is, fault is the actual cause of a failure, and error is just an indication or sign of a fault. Multiple errors could be due to a fault, and even a single error could be the cause of multiple failures. With many independent resources cooperating together as one, the chance of failure of an individual resource increases drastically, particularly if the resources are very physically dispersed and connected using network links. With the possibility of many thousands of computing resources operating together, the odds of a long running process not failing on at least one resource is almost zero [11, 12]. Also, according to Townend and Xu [13], it was posited that as applications scale to take advantage of Grid resources, their size and complexity increase drastically. Those systems with complex asynchronous and interacting activities are very prone to errors and failures due to their extreme complexity. Therefore failure free applications are unfeasible irrespective of the fault avoidance and fault removal techniques implemented [13]. It is more likely that errors will be aggravated by the fact that many Grid applications will perform long tasks that may require several days of computation, if not more.

Most of the scheduling algorithms however, assume that resources are fully reliable and there is no failure of resource while processing group of tasks. The Grid has so many resources, such that the probability of some resources failing is very high. Most often, if any of the allocated resources fails during job execution, the job is rescheduled on another resource which would start executing the same job from scratch. Even though some of these resources satisfy the criterion of deadline constraint, they still have tendencies toward failure. In such a scenario, the scheduler goes ahead to select the same resource for the simple reason that the Grid resource assured to meet user’s requirements of the jobs. This leads to more time being consumed executing the user job than expected. This is a setback to the Grid providers and users. A record number of research contributions, whose efforts ensure the integration of fault tolerant components into scheduling algorithms in the Grid computing environment, do exist [14– 16].

The main goal of this paper is to incorporate a fault-tolerant scheduling mechanism into an ant colony load balancing scheduling algorithm tagged *AntZ*, which was initially proposed by Ludwig and Moallem [8]. However, the specific goal of the paper is to design and implement an efficient fault tolerance algorithm that will:

1. Enable job execution in spite of resource failure in the context of Grid computing environment.
2. Improve user QoS requirements (e.g. deadline to complete job execution).
3. Reduce the selection probability of resources with more fault occurrence history.

Therefore, we intend to achieve the above goal through the:

1. Incorporation of fault tolerant scheduling algorithms into an existing *AntZ* or ant colony optimization (ACO) Grid load balancing algorithm proposed in [8].
2. Implementation and simulation of the proposed scheduling algorithms using GridSim Toolkit simulator.
3. Performance comparison of ACO (*AntZ*) with the proposed method in terms of makespan, average turnaround time, throughput and resource utilization.
4. Statistical analysis of the proposed and existing algorithms’ results with the objective of drawing a rigorous and fair conclusion.

The remainder of the paper is organized as follows. In Section 2, a review of related scheduling algorithms and fault tolerance scheduling mechanisms in Grid environment is carried out. In Section 3, the architecture of the scheduling algorithm is discussed. In Section 4, implementation of a prototype system and performance evaluation of the proposed algorithm is carried out. Section 5 presents concluding remarks and outlines future directions.

## Related work

In [8], two new distributed swarm intelligence inspired load balancing AntZ and Particle Swarm Optimization (PSO) scheduling algorithms are discussed. In the *AntZ* approach or ACO as it is called in this paper, each job submitted to the Grid invokes an ant which searches through the network to find the best node (lightest loaded node) to deliver the job to. Ants leave information related to the nodes they have seen as a pheromone in each node which helps other ants to find lighter resources more easily and also carry the load information of the visited nodes along with themselves. However, the decision making as to which node to take next is either by looking at the load information table of the nodes or they choose a node randomly by the probability of a mutation factor. At the end, the ant delivers the job to a resource and dies. In the PSO approach, as jobs are submitted to the node in the network, they go in a local queue list of jobs in each node waiting for their turn to be executed. Each resource in the network is considered to be a particle and nodes communicate with each other about their load information to find a better candidate to execute their workload. The amount of workload being submitted is being controlled by a threshold defined by the load difference. However, one major limitation of the ACO scheduling algorithm is the lack of resource failure handling, and which is the main goal the proposed work intends to address.

Fault tolerant measures, in Grid environment are different from those of general distributed systems [17]. Fault tolerance is a crucial issue in Grid computing. Also in large-scale Grids, the probability of resource failure is much greater than in conventional parallel systems. Thus, fault tolerance is an area of exploitation in Grid computing. Nazir, et al., [18], avers that with the emergence of Grid computing more emphasis will be on fault tolerance, and that Grid computing will impose a number of unique new concepts and technical challenges to fault-tolerance researchers. The three fault tolerance techniques highlighted in their work include; checkpointing, replication and adaptability. Similarly, in [18], an adaptive fault tolerant job scheduling strategy for Grid scheduling called CFTGS is proposed. The fault tolerant strategy of the CFTGS is checkpointing-based. It maintains the fault index of Grid resources. The scheduler makes scheduling decisions according to the value of resource fault index and response time. Simulation results show that the proposed algorithm is able to execute more jobs successfully within the specified deadline and allotted budget, while improving the overall execution time and minimizing the execution cost of Grid jobs.

Checkpointing is one of the prevalent techniques used to provide fault-tolerance in unreliable systems [5]. It records the snapshot of the entire system state in order to restart the application after the occurrence of a resource failure. It is applied specifically to areas where there are demands for high QoS with respect to non-violation of service level agreement (SLA) between the system users and the resource providers [19]. Checkpointing performs the task of saving the system state into a permanent storage location so as to retain the current state of the system processes in the course of system failure, and as such the system would not have to start the execution process all over from the scratch, but at the last checkpoint read. Usually all checkpoint measurement data collected are stored either on a temporary or stable storage medium. A rollback mechanism is incorporated into the checkpoint, to allow system restore at any state of the running process [20]. Checkpointing strategy can be applied to any system or program that is susceptible to failures. However, other options of fault handling with regards to load balancing in the Grid have been exploited. Next, we briefly discuss the fault tolerant system proposed by Balasangameshwara and Raju [14].

In [14], a fault tolerant hybrid load balancing strategy referred to as AlgHybrid_LB, which takes into account Grid architecture, computer heterogeneity, communication delay, network bandwidth, resource availability, resource unpredictability and job characteristics is proposed. The AlgHybrid_LB juxtaposes the strong points of neighbor-based and cluster based load balancing algorithms. The main goal of this fault tolerant system is to achieve minimum response time and optimal computing node utilization with different job assignments. Experimental results show that the proposed algorithm performs very well in a large Grid environment with drastic reduction in additional communications induced due to load balancing. However, the system is limited by scalability and robustness, based on the two passive replication strategy used for the implementation of the proposed fault tolerant load balancing algorithm. These missing components which our proposed ant colony optimization fault tolerant algorithm incorporates are vital and required for handling faults in a dynamic environment such as the Grid.

With respect to swarm intelligent based fault tolerant systems, Khanli *et al*., [15], proposed a new Genetic Algorithm (GA) that uses resource fault occurrence history (RFOH) to achieve reliable job scheduling in the computational Grid. In this case, the Grid Information Server (GIS) maintains the history of fault occurrence in resources, in a table called fault occurrence history table (FOHT). The FOHT has two columns. First column presents the histories of resources faults, while the second column keeps track of the number of successful job execution by resources. Therefore, the number of rows and the number of resources are equal. The fault index is incremented or decremented depending on the job status. The experimental results show that the proposed strategy is capable of reducing the total job execution time and at the same time decreases the probability of failure, thereby increasing the overall system reliability. Similar application of GA based fault tolerant algorithm with related strategy on fault handling in the Grid system is presented in [20].

## Fault tolerance architecture

**Fig 1:**
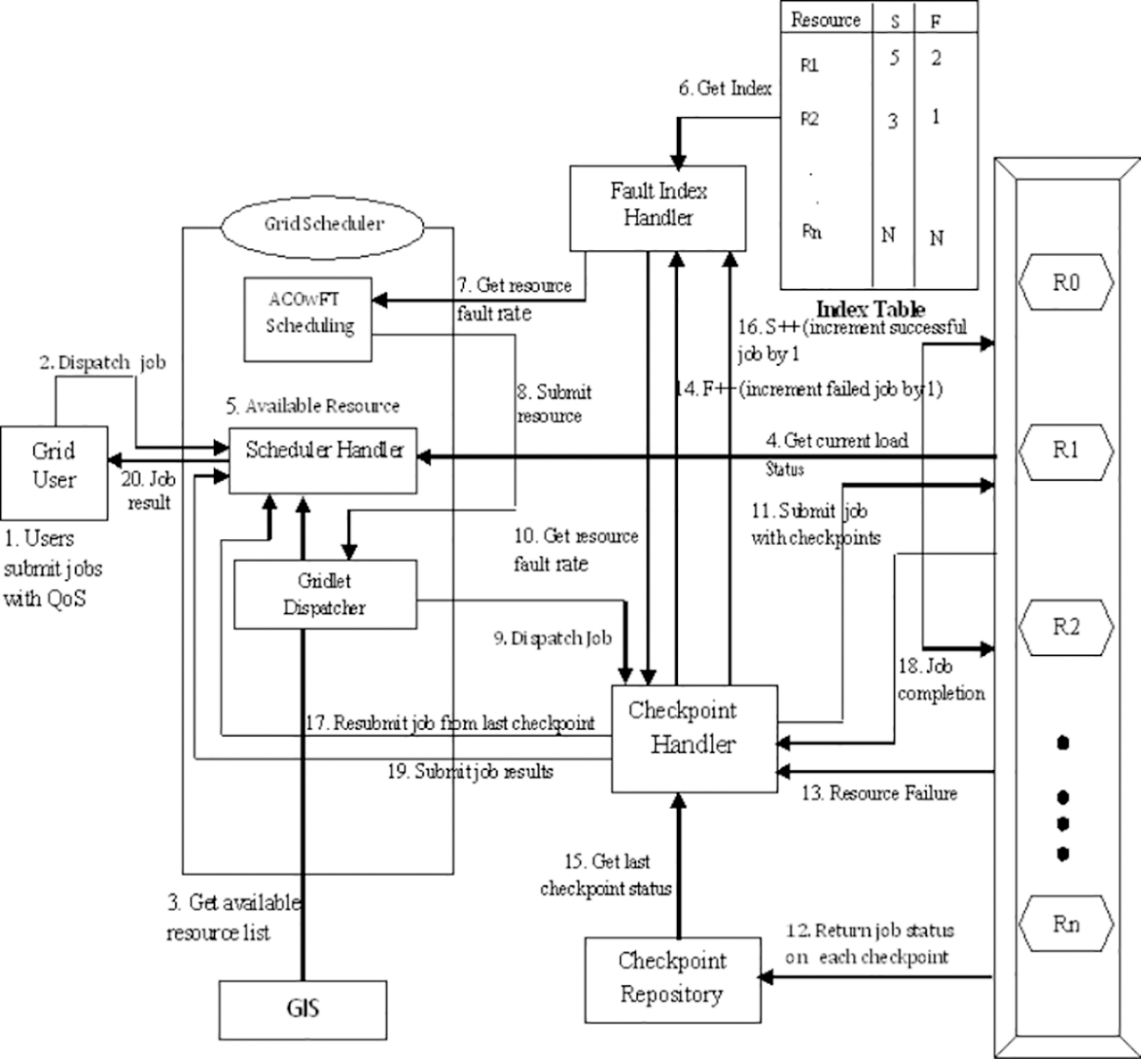
Proposed fault tolerant architecture. The fault index value suggests the rate of tendency of resource failure; the lesser the fault index value, the lesser the failure rate of the resource and the higher the fault index value, the higher the failure rate. Checkpoint handler queries the checkpoint repository to obtain latest checkpoint files of the executed jobs on the failed resource and reschedules the jobs along with last checkpoint status (see Algorithm 7). On the successful completion of the job, the checkpoint handler receives the job completion message from the Grid resource and updates the fault index handler to increment the success rate of the resource The fault index handler maintains a fault index history of the Grid resources, which indicates the failure rate of the resource. To update and maintain the fault index of a Grid resource, the fault index handler uses Algorithm 6 described below to take decision:

An architectural view of the fault tolerant system in Grid environment considered in this paper is depicted in Fig 1. The scenario is as follows: The clients through a user interface submit their jobs to the Grid transparently specifying their QoS requirements such as the cost of computation, deadline to complete the execution, the number of processors, speeds of processing, internal scheduling policy, and time zone. Grid scheduler which is an important entity of the Grid is connected to an instance of a user [18]. Each Grid job is first submitted to its scheduler, which then schedules the Grid job according to the user’s scheduling policy. The scheduler maps jobs received from users to Grid resources. Scheduling decisions taken by the scheduler are based upon information provided by the Grid Information Service (GIS), which contains information about all available Grid resources with their computing capacity and cost at which they offer their services to Grid users. All resources that join and leave the Grid are monitored by GIS. The component Fault Handler is responsible for detecting failure in resources and estimating the required information for fault tolerance process. If the fault handler detects a Grid resource failure during execution of job, the job is rescheduled on another resource which starts executing the job from scratch. This leads to more time consumed executing the job than expected. Thus, the user’s QoS requirements are not satisfied. The scheduling system acts not only as an interface between users and Grid resources but also provides reliable service to users. The pseudo-codes for the AntZ [8] approach are provided in Algorithm listing 1, 2, and 3.

The interaction between the different components of the system is also depicted in Fig 1. When Grid Scheduler receives a Ggrid job from users, it connects to the GIS to get information of available Grid resources and then requests the resources to send their current work load condition and after that it gets the fault rate history of each resource from the fault manager. The scheduler collects the set of appropriate resources along with their load and fault rate and invokes the ACO techniques. The ACO with fault tolerance technique presented in Algorithms listing 4, 5, and 6 are implemented to search for the resource that meets the user’s requirements using the fault rate of the resource and the load on the resource, to make appropriate decisions. A Resource is selected based on the current work load and fault rate of the resources, and before forwarding the job to the selected resource, a job dispatcher dispatches the jobs to the checkpoint handler. On receiving the job from the job dispatcher, the checkpoint handler gets the number of successful jobs completed and number of failed jobs of the resource from the fault index handler on which it is scheduled and sets the number of time to checkpoint and the checkpoint interval based on the failure rate of the resource. The checkpoint handler then submits the job along with the checkpoints to the selected resource. The checkpoint repository receives partially executed result of a task from a Grid resource in the intervals specified by the checkpoint handler. It maintains Grid tasks and their checkpoint table which contains information of partially executed tasks by the Grid resources. For a particular job, the checkpoint repository discards the result of the previous checkpoint, when a new value of the checkpoint result is received. When a particular job is completed, its entity will be removed from the checkpoint result table.

**Algorithm** 1: Pseudocode for the existing *AntZ* algorithm (ACO)

*A user submits a Job to its local* *resource node*

*An Ant is created and invoked in response to the user’s requests*, *the job is delivered to the ant*.

*For each* *Job {*

    *(A)Ant starts to move from node to node for a number of steps searching for the best suitable node (lightest loaded node)*.

      *For each step taken by the ant {*

        *(i) Pheromone Laying*

        *Ant collects load information of the node it is visiting and adds to its history*

        *Updates the load information table in the visiting node*

        *(ii) Decision Making*

        *Look up Load table information of nodes*

        *Random selection with a probability of mutation factor*

      *}//end* *for*

    *(B) Ant deliver job to a particular resource and dies*

*}//End* *for*

**Algorithm** 2: Existing *AntZ* scheduling algorithm (ACO)

1: ***function*** Ant (Gridlet_ID) {

2:  Initialize () // initialization of variables

3:   ***While*** (step < MaxSteps) {

4:    currentNode = LocalResourceID

5:      currentLoad ←getNodeLoadInformation

6:      AntHistory.**add**(currentLoad) // database storage

7:      localLoadTable.update // database storage

8:      ***If*** random() < MutRate ***Then***

9:         nextNode = RandomlyChosenStep //randomly generate number

10:    ***Else*** nextNode = getLighterNodeInHistory()

11:      MutRate = MutRate—DecayRate

12:      step = step + 1

13:     currentNode = nextNode // moveToNextNode

14:} //***End While***

15: deliverJobToNode

16:} //***End*** function Ant

**Algorithm** 3: Existing *AntZ* for acquiring node history (ACO)

1: ***function*** getLighterNodeInHistory(){

2: bestNode ← currentNode

3: bestLoad ← currentLoad

4: ***for*** entry ← 1 to *n* {

5:***If*** entry.load < bestLoad {

6: ***Then*** bestNode ← entry.node

7: **Add**(bestNode)}

8: ***Else If*** entry.load = = bestLoad

9: ***Add***(entry.load)

10:} // end for

11: ***If* Add() > 0**

12: randomlypickFrom ***Add()***

13: ***Else*** bestNode

14:} // ***End*** algorithm

In the proposed scheduling fault tolerant algorithm depicted in Algorithm 4, the ant collects load information of the resources as well as the fault rate of the node it visited and adds it to its memory using Algorithm 5. The job is submitted to the resource with checkpointing status. That is, during execution, the process state will be saved at a particular period.

The Fault Handler in Grid system as shown in Fig 1 monitors the status of the resources available in the Grid at regular intervals. If any failure occurs, it is reported to the checkpoint handler. Checkpoint handler updates the fault index handler to increment the failure index of the Grid resource.

**Algorithm** 4: Proposed ACO with Fault Tolerance algorithm (ACOwFT)

1: ***function*** Ant (Gridlet_ID){

2:  Initialize () // *initialization of variables*

3:   ***While*** (step < MaxSteps) {// *while stopping criteria are not satisfied*

4:     currentNode = LocalResourceID

5:     currentLoad←getNodeLoadInformation(currentNode)

6:      currentFault ← getFaultRate

7:      AntHistory.add(currentLoad, currentFault) //database storage

8:      localLoadTable.update // database storage

9:    ***If*** random () < MutationRate ***Then***

10:     nextNode = RandomlyChosenStep //randomly generate number

11:           **Else** nextNode = getLighterNodeInHistory() 

12:      MutationRate = MutationRate—DecayRate

13:      step = step + 1

14: currentNode = nextNode

15:} //***end while***

**Fig 2:**
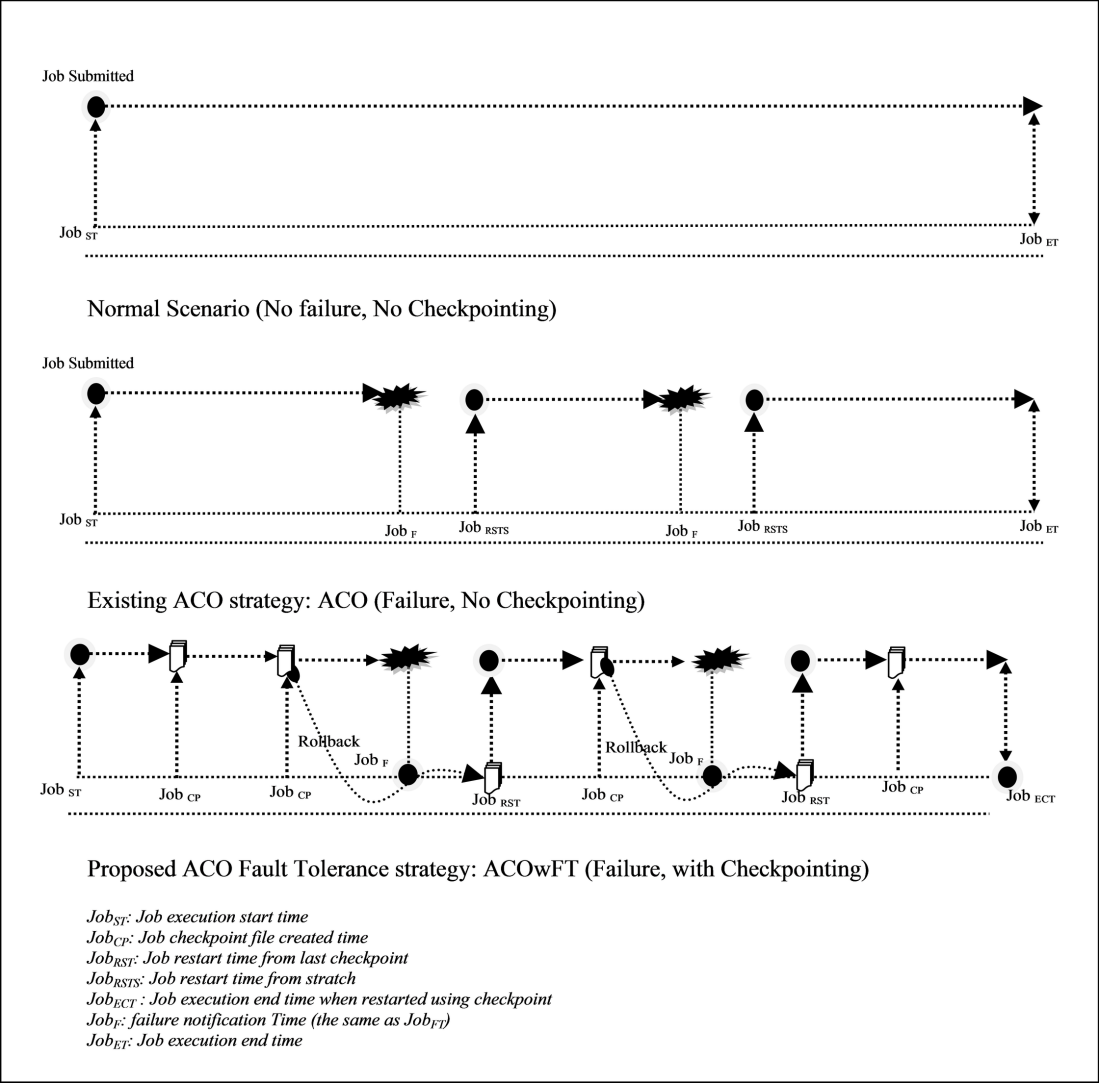
An illustration of the recovery analysis.

16: ***AssignCheckPoint ()*** // *depicted in Fig 2 pseudo code*

17: deliverJobwithCheckPointToNode

18:} //end function

**Algorithm** 5: Proposed ACOwFT algorithm for acquiring node history

1: **function** getLighterNodeInHistory () {

2: bestNode ← currentNode

3: bestLoad ← currentLoad

4: bestFault ← currentFault

5: ***For*** entry ← 1 to *N* {

6; ***If*** entry.load <bestLoad and entry.fault < bestFault ***Then*** {

7:        bestNode ← entry.node

8:        bestLoad ←entry.load

9:        bestFault ← entry.fault

10:      ***Add*** (bestNode)}

11: ***Else If*** entry.load < bestLoad and entry.fault > bestfault ***Then*** {

12:      bestNode ← entry.node

13:        bestLoad ←entry.load

14:        bestFault ← entry.fault

15:       ***Add***(bestNode)}

16: ***Else If*** entry.load = = bestLoad and entryfault = = bestfault ***Then***{

18:        add(entry.node)}}

19: ***If Add***() > 0

20: randomlypickFrom ***Add***() 

21: ***Else*** bestNode

22:} // *End* for

23:} // *End* funtion

**Algorithm** 6: ACOwFT algorithm for fault index handler

***1*.    *If*** the checkpoint handler receives the job completion message from resource ***Then***

      •    Send a message to the fault index handler to increment success index of the resource.

      •    Submits finished job to the scheduler.

    ***End If***

*2*.    *If* the checkpoint handler receives the failure message from the resource monitor

    ***Then***

      •    Send a message to the fault index handler to increment the failure index of the resource that fails to complete the assigned job.

      •    Send a message to the checkpoint repository, to check checkpoint status of this job.

***3*.    *If*** the checkpoint result of the job exists in the checkpoint repository

    ***Then***

      •    Submit the last checkpoint data received, to the scheduler handler for rescheduling.

      •    Exit

    ***End If***

***4*.    *If*** the checkpoint result of the job does not exist in the checkpoint repository

    ***Then***

      •  Resubmit the job to start from scratch to the scheduler handler for rescheduling.

      •  Exit

    ***End If***

     ***End If***

**Algorithm** 7: Pseudocode for ACOwFT Checkpointing

  1: set checkpoint time and interval for a particular job

  2: submit the job for execution

  3: ***While*** (job is running till the end)

  4:    ***If*** currentTime > = checkpointTime ***Then***

  5:        Update checkpoint interval time

  6:        Increment number of time checkpoint

  7:        Checkpoint current process state

  8:    ***Else***

  9:        not time for checkpoint

  10:    ***End***

The variable initialization function for the algorithm 4 as used in the experimental setup is given as follow:

***Initialize ()***

**{**

        Ant_ID = Gridlet_ID

        ResourceID = IDofCurrentResource;

        Initial resourceLoadInformation = getResourceLoad; //Gridsim compute initial pheromone

        Initial resourceFault = getResourceFaultRate;

        Wander_Number = 5; // MaxStep

        MutationRate = 0.5;

        DecayRate = 0.2;

        AntIsFinish = false

**}//End of** **Initialize**

### Checkpointing implementation

Checkpointing is a combination of two activities, first, it saves the running data and restores it after getting a suitable resource. Second, it captures the states and data of a running process including registers containing the address, variables, libraries, data structures, files containing data with a large size, etc. An application-level checkpointing tool is adopted, which is directly implemented within the application source code. That is, the application contains source code that saves and restores critical program variable to and from stable storage.

The checkpointing of an object-oriented program is considered, in which the state of the program can be recovered from the contents of the fields of the objects. In this context, checkpointing amounts to recursively traversing the objects and recording the local state of each one. It is essential to identify what is to be checkpointed. The implementation consists of a GridletCheckpointing, which specifies the logical condition that must be provided by each object to be checkpointed. When it is time to take a checkpoint, the application writer is expected to explicitly call the function CheckpointRepository() whenever a checkpoint is required. GridletCheckpointing object drives the checkpointing process.

The checkpoint file used in this experiment is a Hash function. Hashing is a technique of storing values and searching for them in tables. Table is a set of table entries, (K; V), each containing a unique key K, and a value (information) V. Each key uniquely identifies its entry. For simplicity the assumption made is that checkpoints are written to this hash table. Each checkpoint contains only the pages that have been modified since the previous checkpoint. Here, Hash table keyed is used, to keep track of the necessary objects. It uses a hash table keyed on identity hash code of the objects. The hash table maps from the object to a pair: <object, address>. Object is referenced back to the object; address refers to the address of the object in the output image.

### Rollback recovery analysis

In a conventional system, when a failure occurs, usually a job is rescheduled on another Grid resource and execution start from the beginning. A technique to avoid restarting the application from the beginning is the rollback recovery, which is based on the concept of checkpointing. The checkpointing mechanism periodically saves the application’s execution state to stable storage. Hence, whenever a failure interrupts a volunteer computation, the job can be resumed from its last successful state, thereby improving the QoS requirement of the users.

An illustration of the fault tolerance scheduling algorithms is depicted in Fig 2. The first diagram shows a normal environment where job failure does not exist, when the job is submitted it run to completion. Although with the Grid environment where resource failure is guaranteed, the dynamism is put into consideration. The scenario is as follows: In the ACO algorithm (Algorithm 1), when a resource failure occurs, the scheduler needs to reschedule the job which starts its execution from scratch, leading to an increase in the execution time of the job. To improve on the performance of the algorithm presented in [8], a checkpoint based fault tolerance and recovery strategy is integrated into the existing algorithm, which is called ant colony optimization job scheduling with fault tolerance (ACOwFT) in Grid Computing by using checkpointing mechanism, the return time will be improved considerably, this is useful for environments with high source fault rate [21].

In the new proposed ACOwFT algorithms (see Algorithm 4), the job execution status is preserved in the form of checkpoints. When a resource failure occurs, the job is restarted exactly from the last saved checkpoint, thereby eliminating the need to start executing the job from scratch again. Hence, the execution time of the job is reduced. As presented in [15], the submitted job starts its execution at time *Job*_*ST*_ and finishes its execution at time *Job*_*ET*_ in a normal environment, that is, the execution time of the job *ET* is given in Eq 1.

$${ET=(Job}_{ET}-{Job}_{ST})$$ (1)

And without considering checkpointing, if failure occurs at time *Job*_*FT*_ then the job restarts its execution from the beginning at time *Job*_*RSTS*_ and finishes at time *Job*_*ET*_, that is, the Total Execution Time (*TET*) of the job as given in Eq 2 is the summation of the following: *Job*_*FT*_—*Job*_*ST*_*; Job*_*FT*_—*Job*_*RSTS*_*; Job*_*ET*_*—Job*_*RSTS*_*;*

$${TET=(Job}_{FT}-{Job}_{ST})+({Job}_{RSTS}-{Job}_{FT})+({Job}_{ET}-{Job}_{RSTS})$$ (2)

If the job execution status is maintained in the form of a checkpoint file, and the last created checkpoint is at time *Job*_*CP*_ which is before *Job*_*F*_, then the job can be restarted from the last checkpoint at time *Job*_*RST*_. Also the total completion time as given in Eq 3 will be the summation of *Job*_*FT*_—*Job*_*ST*_*; Job*_*FT*_—*Job*_*RST*_*; Job*_*ET*_*—Job*_*RST*_ (Fig 2).

$${TET=(Job}_{FT}-{Job}_{ST})+({Job}_{FT}-{Job}_{RST})+({Job}_{ECT}-{Job}_{RST})$$ (3)

### Checkpoint interval

The efficiency of a checkpoint mechanism is strongly dependent on the length of the check pointing interval. Checkpointing interval is the duration between two checkpoints. Each interval starts when a checkpoint is established and ends when next checkpoint is established. Frequent checkpointing leads to a large number of redundant checkpoints, which may enhance overhead like delay job processing by consuming computational and network resources. On the other hand, lazy check pointing may lead to loss of significant computation because a substantial amount of work has to be redone in case of a resource failure [22]. Hence, the decision about the size of the checkpoint interval and the checkpoint technique is a complicated task and should be based upon the knowledge about the application as well as the system [23].

Most utilized checkpointing mechanisms use resource fault index to determine checkpointing interval. It was shown in [24], that resource failure rate is more effective than resource fault index. Resource failure rate is used to represent the failure history of a resource. So, the resource failure rate (*FR*) is used to determine the checkpoint interval and the number of checkpoints instead of using the resource fault index [24]. Eq 4 calculates the fault rates of the resources [25] and Eq 5 calculates the number of times to checkpoint a particular job when it is running and Eq 6 calculates the checkpoint interval time when a job should be checkpointed.

$$FR=\frac{N_{f}}{N_{s}+N_{f}}$$ (4)

Where *N*_*f*_ is the number of failed jobs assigned to the resources and *N*_*s*_ is the number of job submitted to the resources.

$$CheckpointNumber=R_{t}*FR$$ (5)

*where R*_*t*_ = *Response time*

$$checkpointInterval=\frac{R_{t}}{R_{t}*FR}$$ (6)

A resource is considered to have failed, when a resource service stops due to resource crash or the resource is withdrawn from the Grid system. The checkpoint handler interacts with the scheduler to perform the rescheduling of a job unconditionally, using a checkpoint. Therefore, the main goal of Algorithm 4 is to minimize job processing time, improve throughput, and to also consider the failure rate of resources during resource selection.

## Experimental configuration

For experimental purpose, the resources are assumed to be homogeneous so as to allow efficient comparison between the proposed ACOwFT and existing ACO algorithms, despite the existence of heterogeneous characteristics among Grid resources. Similarly, for experimental purposes, it is likewise assumed that the Grid consists of one computing node (machine) per resource, two Processing Elements (PE) and also network bandwidth speed among the computing nodes as shown in Table 1.

**Table 1:** Grid resource characteristics.

Number of machines per resource	**1**
Number of PE per machine	2
PE ratings	50 MIPS
BandWidth speed	5000 B/S

### Application model

For the application model, we assume that jobs which are submitted to the Grid are independent tasks with no required order of execution predefined priorities. The jobs are of the same computational size, same input files size requirements. The computational requirement (or job length) of each job is presented in Millions of Instructions (MI). In GridSim, jobs are created and their requirements are defined through Gridlet objects [26]. A Gridlet is a package that contains all the information related to a job and its execution management details such as the job length (in MIs), the size of input files, and the job originator (user information). The characteristics of the Gridlet sent to the Grid to compare the performance of different algorithms are shown in Table 2.

**Table 2:** Gridlet characteristics.

Length	**0–50000 MI**
Input file size	100 + (10% to 40%)
Output file size	(10% to 50%)

Since parameter selection may significantly influence the final results obtained for each algorithm performance, the parameter settings for all the simulations conducted on the two algorithms are presented in Table 3.

**Table 3:** Parameterization of the ACOwFT and ACO.

ACOwFT		ACO
Parameter	Values	Values
Number of resources	3,050	100
Number of Gridlets	3,000	1,000
Ant wander number	5	4
Ant mutation rate	0.5	0.5
Ant decay rate	0.2	0.2

### Performance metrics

In this section, performance evaluation criteria which are used to evaluate the performance of the proposed algorithm are defined. The criteria include makespan, average turnaround time, and throughput. As the Gridlets and topologies are generated randomly, although every simulation yields roughly the same result, each single simulation is different from another one; thus, an average of 1,000 runs were made using batch file in order to simulate realistic conditions.

Makespan is defined as the execution time spent from the beginning of the first job to the end of the last job in the schedule. It is assumes that the jobs are ready at time zero and resources are continuously available during the whole scheduling. Then the makespan is obtained by Eq 7:

$$Makespan\;{ET}_{max}=max\{{ET}_{1},\;{ET}_{2},\ldots ,{ET}_{n}\}$$ (7)

where, *ET*_*i*_ is the completion time of job *i*. The lesser the value of the makespan, the more efficient is the algorithm, that is, less time is taken to execute the algorithm.

Another important metrics that is required to measure the efficiency of the new system is the throughput. Throughput is one of the most important standard metrics used to measure the performance of any fault tolerant systems [27, 28]. Here, throughput is defined as Eq 8:

$$Throughput=\frac{n}{TET}$$ (8)

where *n* is the total number of jobs submitted and *TET* is the total execution time necessary to complete n jobs. The throughput metrics is also used to measure the ability of the Grid system to accommodate jobs.

The last metrics considered in this paper is the throughput. Let the total number of jobs be *n*, the completion time for the specified job be *ET* and job arrival time is denoted by *ST*. The turnaround time is defined as Eq 9:

$$Turnaround\;Time={Job}_{ET}-{Job}_{ST}$$ (9)

The average turnaround time is equally defined as Eq 10:

$$Average\;Turnaround\;Time=\frac{\sum_{i=1}^{n}Turnaround\;Time}{n}$$ (10)

Where

N=numberofgridletsandgridlets=jobs

In a scenario where failure is considered for Turnaround Time, Eq 8 will not hold. Therefore Eq 2 was used in the existing algorithm simulation since no fault mechanism was considered. Once a failure occurs, job execution will starts again from scratch. For the new algorithm presented in this paper, Eq 3 was used and when failure occurs, the process will continue executing the job from the last checkpoint recorded.

## Results and discussion

This section shows a comparison of the simulated algorithms when using ACO with Fault Tolerance (ACOwFT), which is the proposed algorithm and the existing ACO without the fault tolerance algorithm for scheduling jobs in distributed Grid environment. In the ACO algorithm, when a resource failure occurs, the scheduler needs to reschedule the job and the job execution usually will start from scratch, leading to an increase in the execution time of the job. But by incorporating Fault Tolerance into the ACO algorithm and that is using the ACOwFT, the job execution status is preserved in the form of checkpoints. Therefore, when a resource failure occurs, the job is rescheduled to resume execution exactly from the last saved checkpoint, thereby, eliminating the need to start executing the job from scratch. Hence, the execution time of the job is reduced. The ACOwFT algorithm was implemented using GridSim simulator to verify the improvement of the proposed approach over the existing ACO algorithm discussed in [8]. The experimental results are analyzed based on performance criterions used.

In order to evaluate the performance of the proposed algorithm, a set of experiments were conducted to measure makespan, average turnaround time and throughput. The result of the proposed scheduling algorithm with Checkpoint (or with failure) is compared with the algorithm in [8], which is without failure control mechanism. In the simulation, scheduling experiments is performed by keeping the resource constant and setting different values to the number of jobs; the number of Gridlet is varied from 100 to 3,700 and the length of each Gridlets is 200,000 MIs at each step. For each job submitted, an Ant is created and ant is assumed to travel from one resource to another in an increment of one (1) time step, each Ant is assumed to take 5 steps and stop when this criterion is reached. It is clear that the efficiency is improved, since the tour of the Ants consider both the resource load and fault rate of the resource. This increases the chance of each Ant to find a resource with low fault tendency.

**Fig 3:**
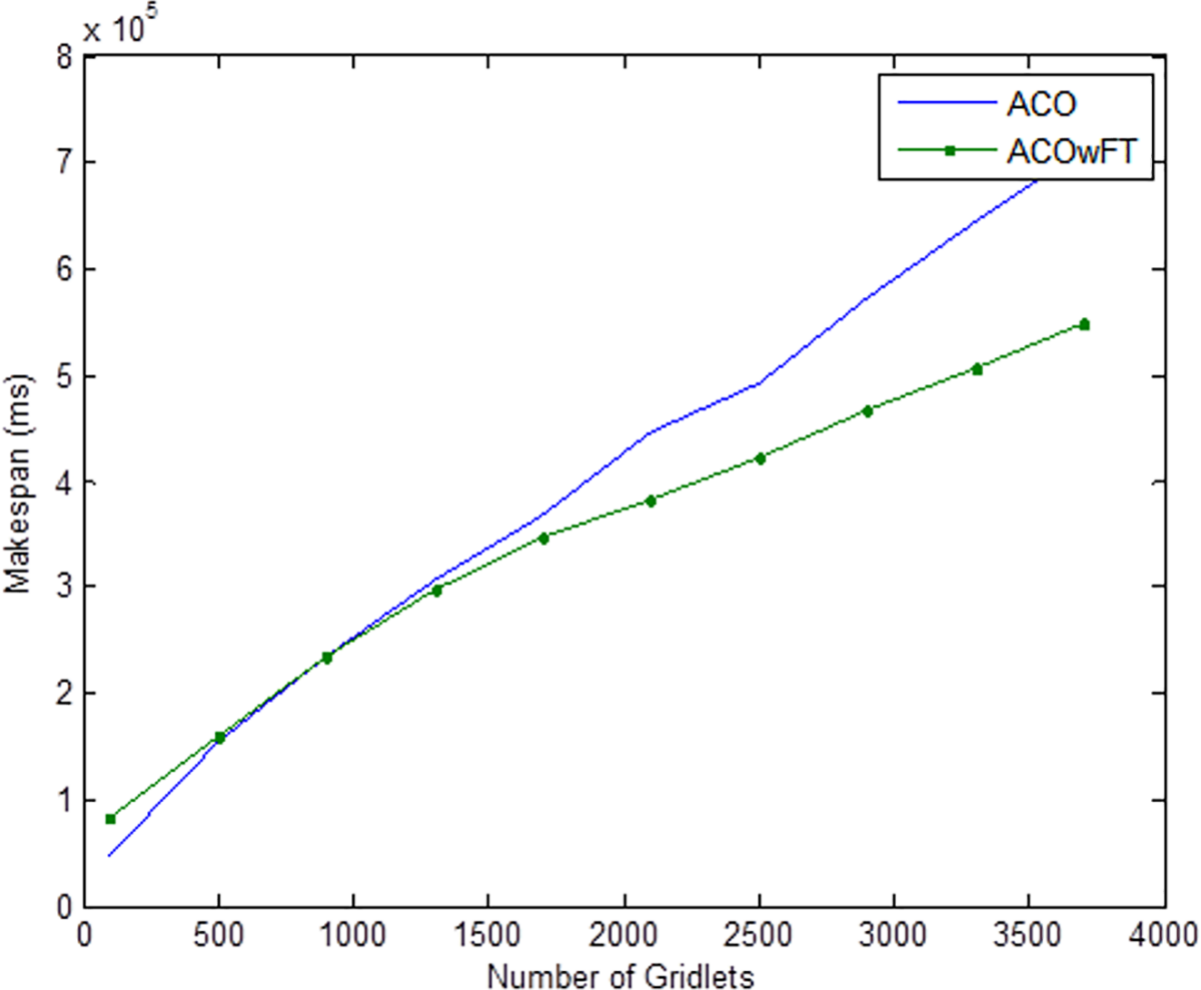
Average makespan for varied Gridlets.

**Fig 4:**
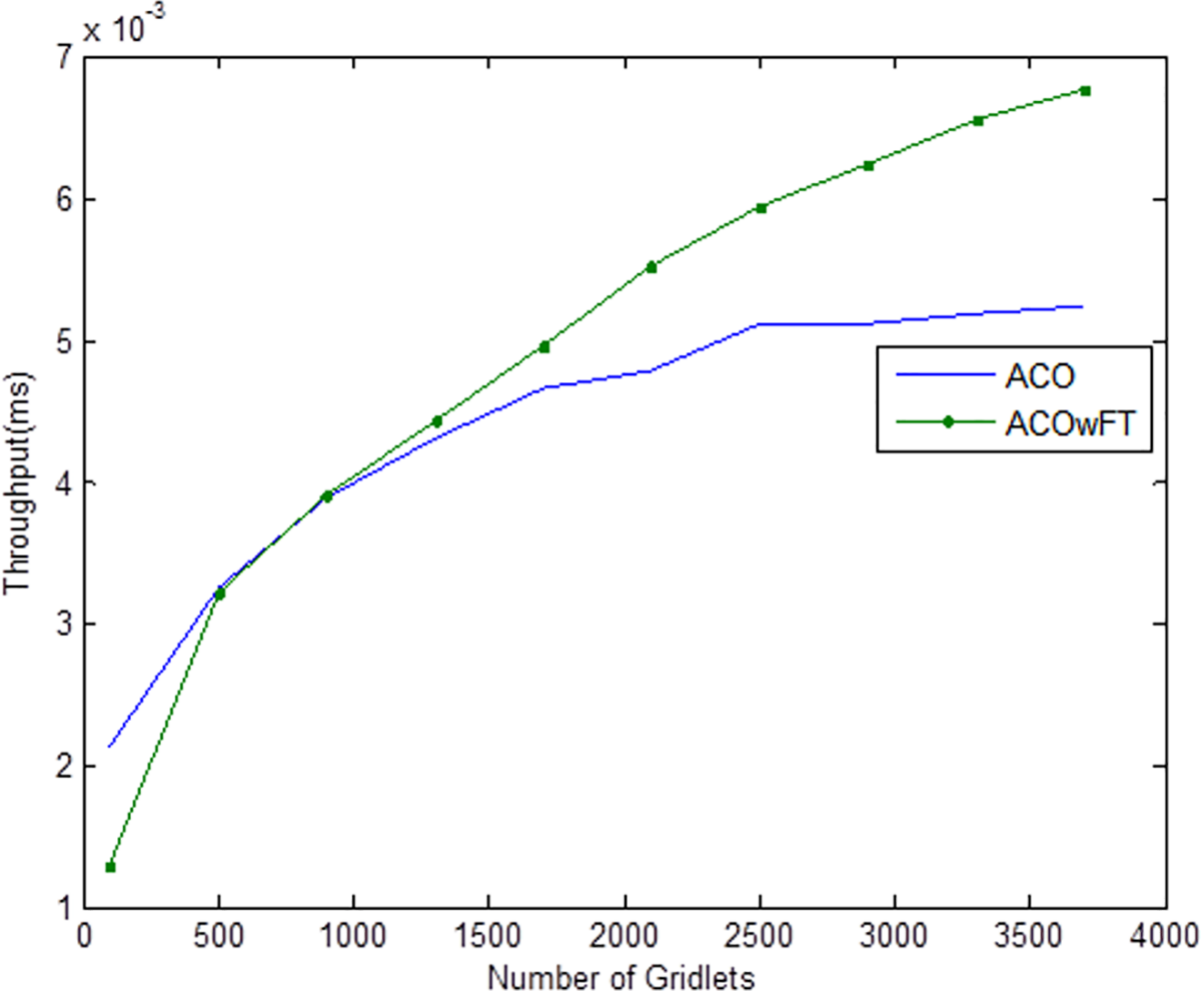
Average throughput for varied Gridlets.

**Fig 5:**
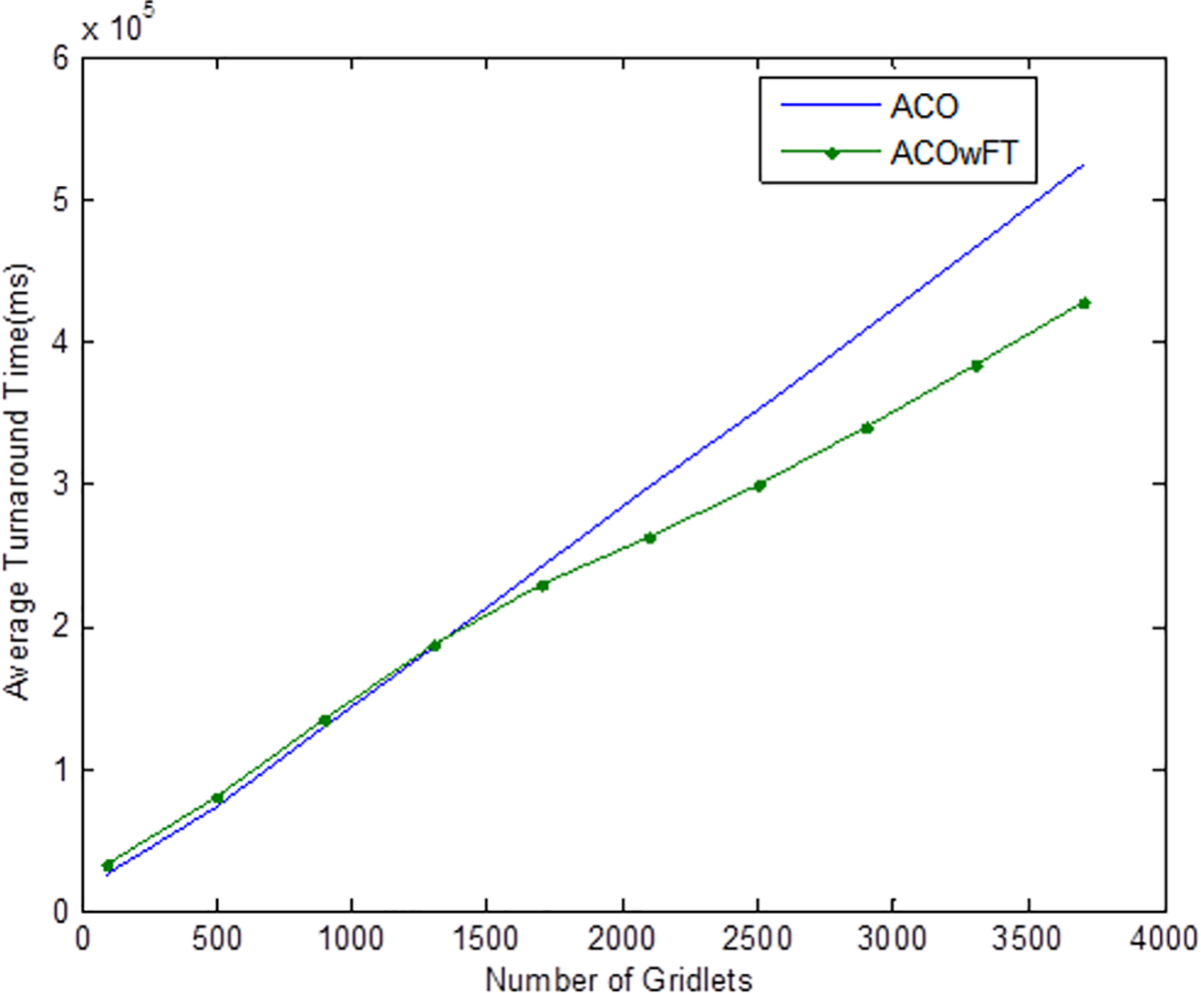
Average turnaround time for varied number of Gridlets. Similar experiments were carried out by keeping the number of Gridlets constant and varying the number of resources. Figs 6, 7 and 8 gives the results obtain as the number of Gridlets is kept constant with varied number of resources. In this experiment, 3,000 jobs are sent to the Grid with varying number of resources from 50 to 3,050, and as can be seen, increasing the number of resources has a decreasing exponential effect on the execution time. The proposed algorithms perform better when there is a small number of resources and a large number of jobs.

Tables 4, 5 and 6 show the simulation results for ACOwFT and ACO algorithms. Figs 3, 4 and 5 depict the makespan, Average Turnaround Time and Throughput. Generally, when there is a decrease in makespan and average turnaround time and an increase in Throughput, we say that there is an improvement of the algorithm over an existing one. With the existing algorithm, as the size of the number of Gridlets is increased, the numbers of jobs failure increases because a particular resource at the time of failure might have more jobs in execution when it failed. The intention here, is to find its effect on the makespan (see Fig 3), as the number of Gridlet increases the performance of the proposed algorithm tends to be better, this is expected since the proposed scheduling algorithms keeps track of the process state of the entire job executing in each resource at a particular time interval. It tends to save a lot of execution time by avoiding the situation of starting all over from the scratch, because having more than one job executing concurrently and when there is a resource failure, it affect the whole jobs in that resource.

**Fig 6:**
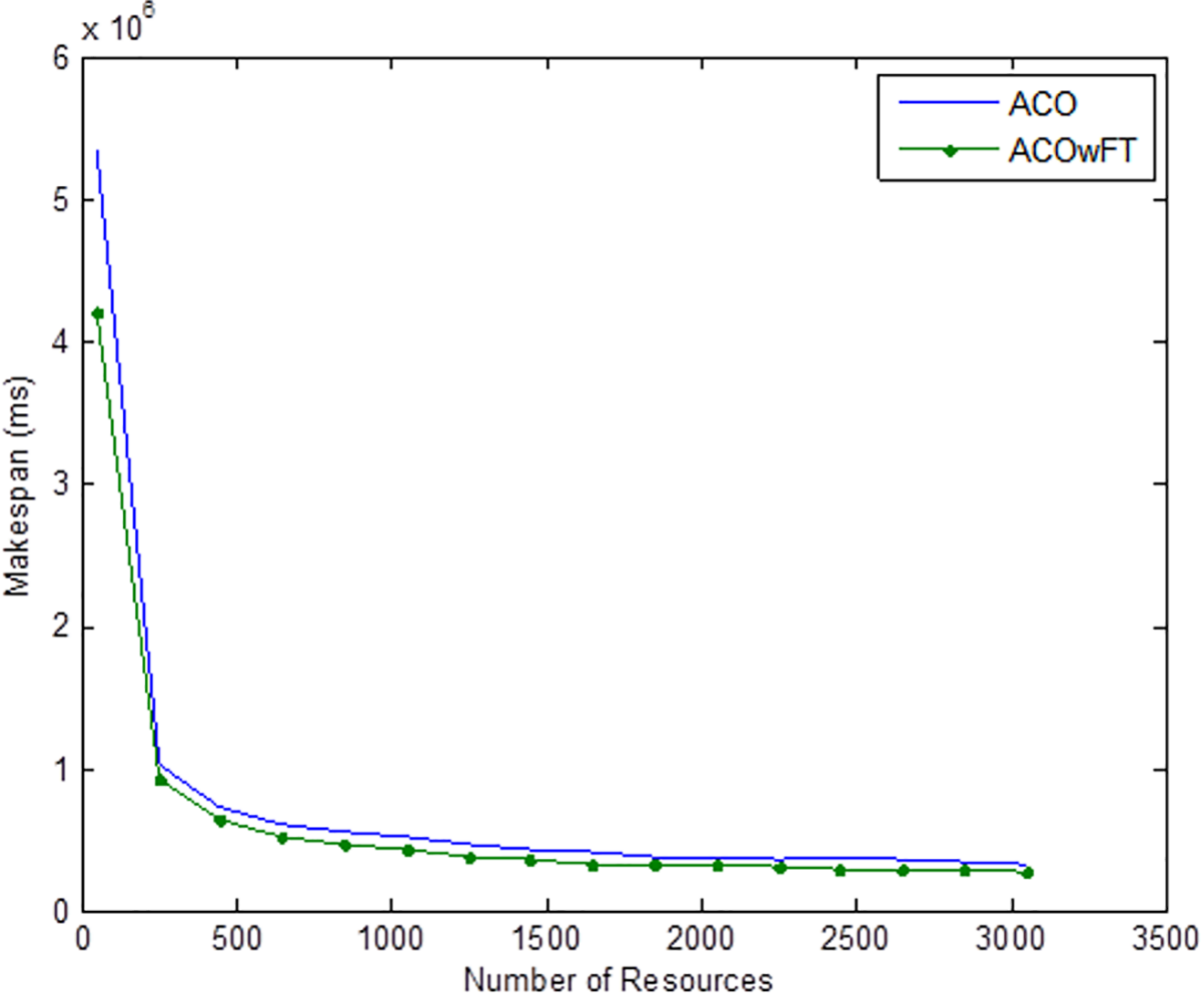
Average makespan time for varied number of resources.

**Fig 7:**
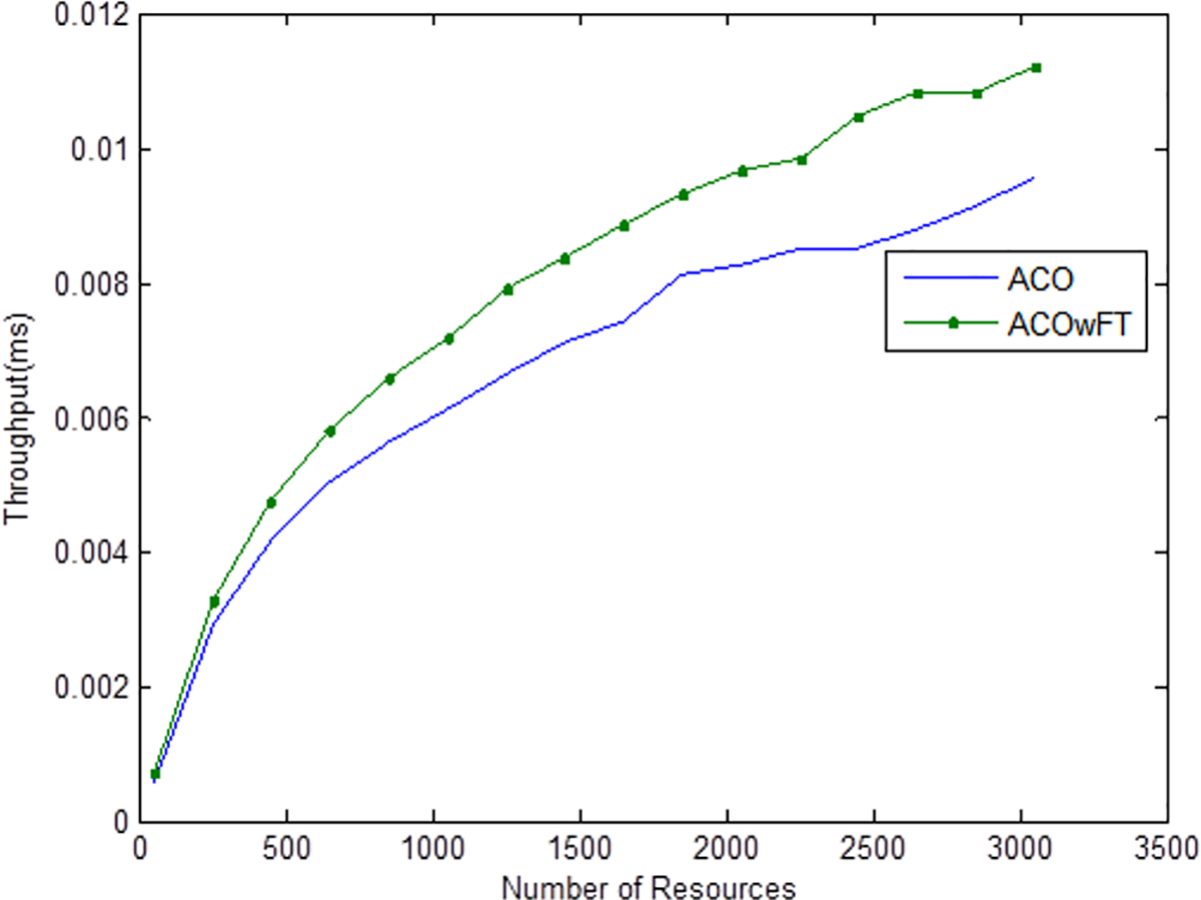
Average throughput time for varied number of resources.

**Fig 8:**
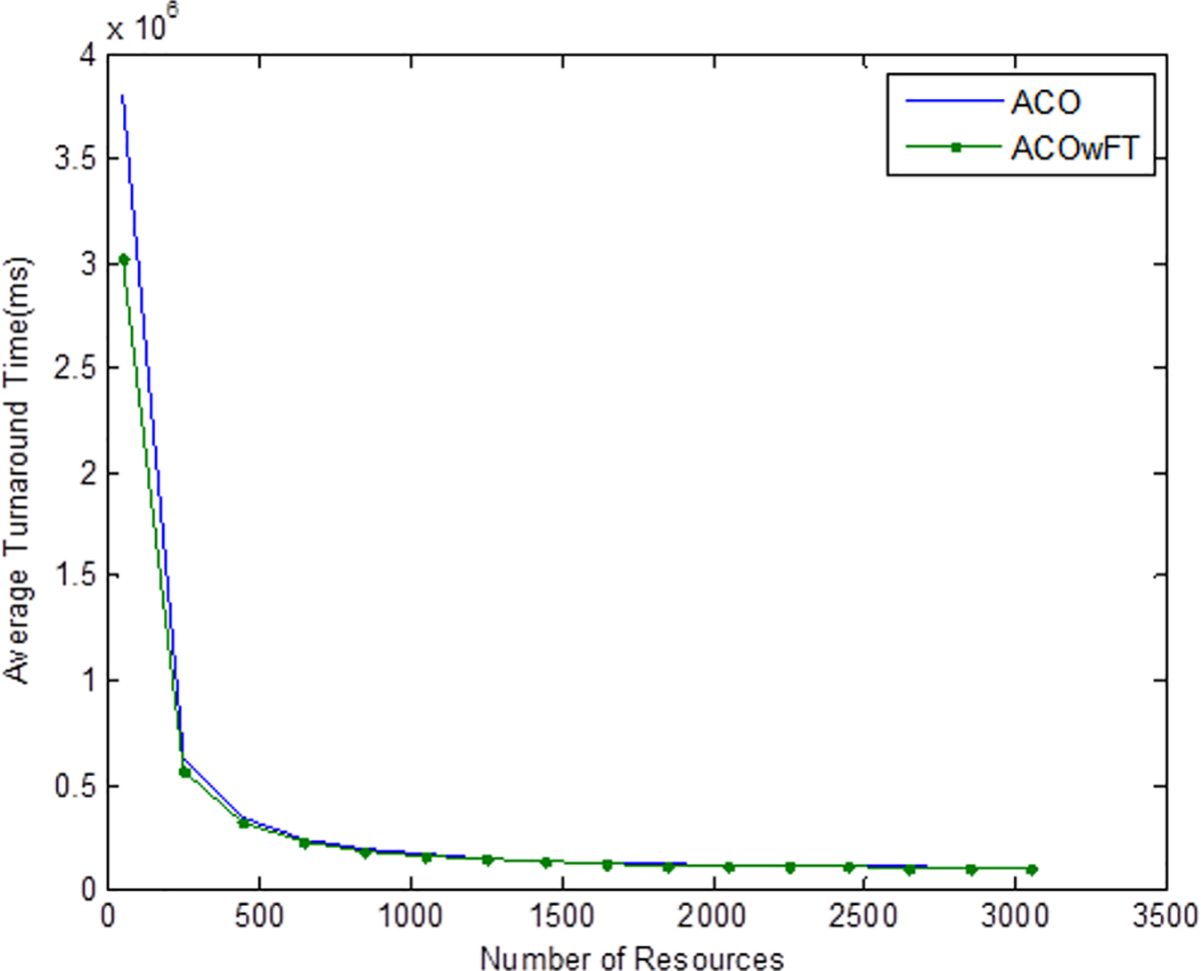
Average turnaround time for varied number of resources. Another important factor that is worth mentioning here is the robustness of the proposed algorithm in comparison with the existing ACO or *AntZ* algorithm. Robustness in this case implies the capability of an algorithm to deal with resource failure when it occurs in the system and to be able to automatically recover from such failure. Since the main goal of the proposed work is to model a fault tolerant algorithm, then a simulation test is further carried out to verify our claim that the proposed ACOwFT algorithm is more robust than the existing ACO algorithm. For this experiment, we considered the case, where 3,000 jobs are sent to the Grid for execution and different percentages of faults deliberately introduced into the system. Similar to the work presented in [24], were the injected fault percentages are assumed from 10% to 50%, in this paper the assumed fault percentages introduced into the system is from 10 to 70%. The essence of introducing a very high fault percentage is to thoroughly evaluate the robustness of the proposed scheduling system under heavy faulty conditions.

**Table 4:** Average makespan table for varied Gridlets.

No. of Resources	No. of Gridlets	ACO [8]^**a**^	ACOwFT^b^
**100**	100	48,569.8266	82,581.19148
**100**	500	156,705.023	159,281.0954
**100**	900	235,171.492	235,238.1332
**100**	1,300	306,692.039	298,183.8147
**100**	1,700	368,990.225	347,092.3771
**100**	2,100	445,146.758	383,579.4808
**100**	2,500	493,102.208	423,205.2016
**100**	2,900	572,050.794	467,180.1945
**100**	3,300	643,413.86	505,964.9605
**100**	3,700	712,712.996	548,524.3321

^a^. The existing Ant Colony Load Balancing: AntZ (ACO)

^b^. The proposed Ant Colony Load Balancing: AntZ with fault tolerance (ACOwFT)

**Table 5:** Average throughput table for varied Gridlets.

**No. of Resources**	**No. of Gridlets**	**ACO**	**ACOwFT**
100	100	0.002137812	0.001295921
100	500	0.003261955	0.003220209
100	900	0.003888949	0.003908796
100	1,300	0.004304247	0.004431326
100	1,700	0.004655346	0.004955437
100	2,100	0.004779502	0.005525678
100	2,500	0.005112216	0.005947022
100	2,900	0.005121511	0.006234242
100	3,300	0.005176597	0.0065532
100	3,700	0.00523709	0.00676183

**Table 6:** Average turnaround time for varied Gridlets.

**No of Resources**	**No. of Gridlets**	**ACO**	**ACOwFT**
100	100	25,191.8559	32,851.01982
100	500	72,079.09801	79,757.59004
100	900	128,938.2533	134,389.9038
100	1,300	185,247.1481	186,865.8473
100	1,700	241,360.751	228,997.5128
100	2,100	297,892.2344	262,358.6086
100	2,500	352,209.5551	298,553.1052
100	2,900	407,783.3016	339,709.521
100	3,300	466,519.0582	383,153.9393
100	3,700	523,189.5081	427,488.4148

In Fig 4, as the number of Gridlets increases, the proposed scheduling algorithms tend to have better result. At the initial state, when the number of Gridlets is 100, the existing algorithm scores a good result, even when Gridlets number is increased to 500, this is because, with few jobs and many resources, resources might be executing few jobs when failure occurs. As the number of Gridlets increases from 500 to 900, the two graphs overlap when the number of Gridlets is at 900 and also at 1,300. As the size of Gridlets increases, the job failure increases because a particular resource at the time of failure might have more jobs in execution when it failed. The proposed scheduling algorithm tends to improve better than the existing algorithm, with a percentage difference of 13%, which is a decrease in the makespan. Fig 4, which depict throughput for varied Gridlets, the goal here is maximization, as Gridlets size increases the graph produces a better result, at size 100 and 500 existing algorithm shows better performance, this is as a result of few jobs executing at that time, at size 900, the two lines in the graph intercepts and also when the size of Gridlets is at 1,300. The graph widens apart as the sizes of the Gridlets increases. From Fig 4, increment in the Gridlets size improved the new proposed algorithm with a percentage increase of 12% when compared with the existing algorithm. 12% decrease in Average Turnaround Time was observed when compared with the same simulation result, as shown in Fig 5. The size of the Gridlets improves the Average Turnaround Time of the proposed scheduling algorithms. This improvement was as a result of checkpointing and selection of resources with low failure tendency.

Tables 7, 8 and 9 gives the results obtain as the number of Gridlets is kept constant and the number of resources varied. In this experiment, 3,000 jobs are sent to the Grid with varying number of resources from 50 to 3,050. Fig 6, which depicts the makespan result simulation, since the size of Gridlets is constant for all resources. As there is an increase in resources, it can be observed that there is a decreasing exponential effect on the execution time. The algorithm achieved 18% reduction in makespan, 18% maximization in throughput and up to 14% reduction in average turnaround time.

**Table 7:** Average makespan time for varied resources.

**No. of Resources**	**No. of Gridlets**	**ACO**	**ACOwFT**
50	3,000	5,333,758.145	4,193,551.119
250	3,000	1,032,621.551	915,672.9539
450	3,000	735,204.4272	635,026.9101
650	3,000	608,917.605	522,718.7224
850	3,000	547,930.762	462,654.4447
1,050	3,000	510,076.0521	425,486.6746
1,250	3,000	471,787.1408	384,175.1397
1,450	3,000	437,496.6908	366,323.4776
1,650	3,000	416,411.9805	324,779.3881
1,850	3,000	384,378.3534	330,142.321
2,050	3,000	379,767.6597	317,569.9529
2,250	3,000	367,627.454	311,514.3109
2,450	3,000	371,217.0904	293,752.8611
2,650	3,000	357,757.9686	282,355.1981
2,850	3,000	341,346.5343	284,203.798
3,050	3,000	330,936.4383	272,435.0624

**Table 8:** Average throughput time for varied resources.

**No. of Resources**	**No. of Gridlets**	**ACO**	**ACOwFT**
50	3,000	0.000578341	0.000723232
250	3,000	0.002929033	0.003296531
450	3,000	0.004154446	0.004769876
650	3,000	0.005048586	0.005824279
850	3,000	0.005629315	0.006571081
1,050	3,000	0.006114395	0.007189014
1,250	3,000	0.00665545	0.007918572
1,450	3,000	0.007119166	0.008371468
1,650	3,000	0.007432594	0.008859062
1,850	3,000	0.008134158	0.009326724
2,050	3,000	0.008270621	0.009682522
2,250	3,000	0.008510047	0.009844879
2,450	3,000	0.00849208	0.010470581
2,650	3,000	0.00878381	0.010836466
2,850	3,000	0.009138704	0.010826396
3,050	3,000	0.009544452	0.011195351

**Table 9:** Average turnaround time for varied resources.

**No. of Resources**	**No. of Gridlets**	**ACO**	**ACOwFT**
50	3,000	3,807,268.06	3,026,939.176
250	3,000	631,508.8115	569,764.0766
450	3,000	345,302.0931	324,526.2434
650	3,000	243,015.8456	232,252.3372
850	3,000	193,772.7683	186,910.5638
1,050	3,000	164,348.6584	159,492.8966
1,250	3,000	145,337.533	141,939.9795
1,450	3,000	133,213.1211	130,883.2574
1,650	3,000	123,799.4381	121,695.203
1,850	3,000	118,449.8406	116,649.3752
2,050	3,000	113,883.6945	112,504.8401
2,250	3,000	110,544.2414	109,215.7295
2,450	3,000	108,441.4055	107,411.0636
2,650	3,000	106,048.3362	104,947.0919
2,850	3,000	104,075.5757	103,099.5684
3,050	3,000	102,759.8428	101,897.4044

**Fig 9:**
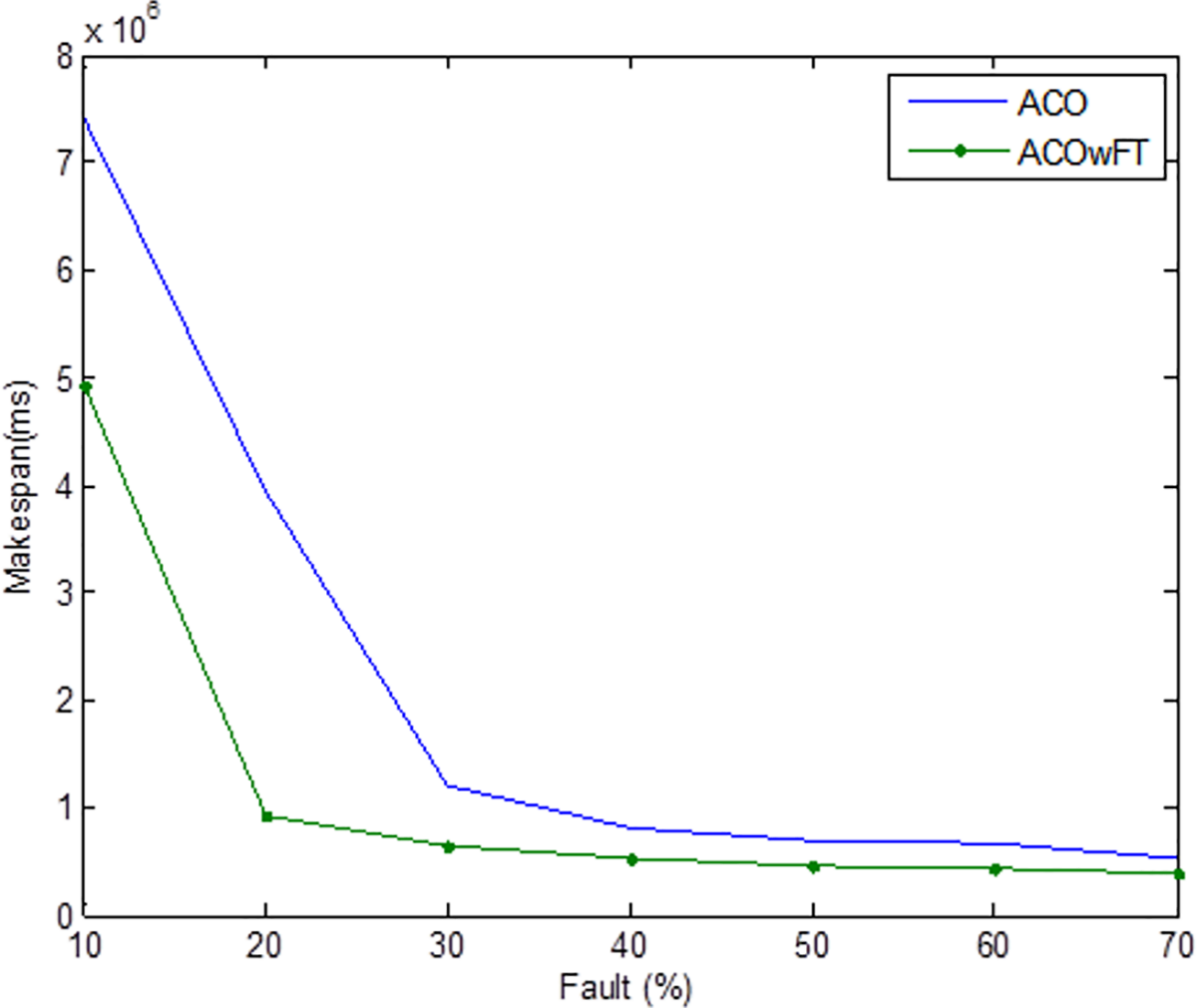
Average makespan time for varied number of faults (total number of Gridlets = 3,000).

**Fig 10:**
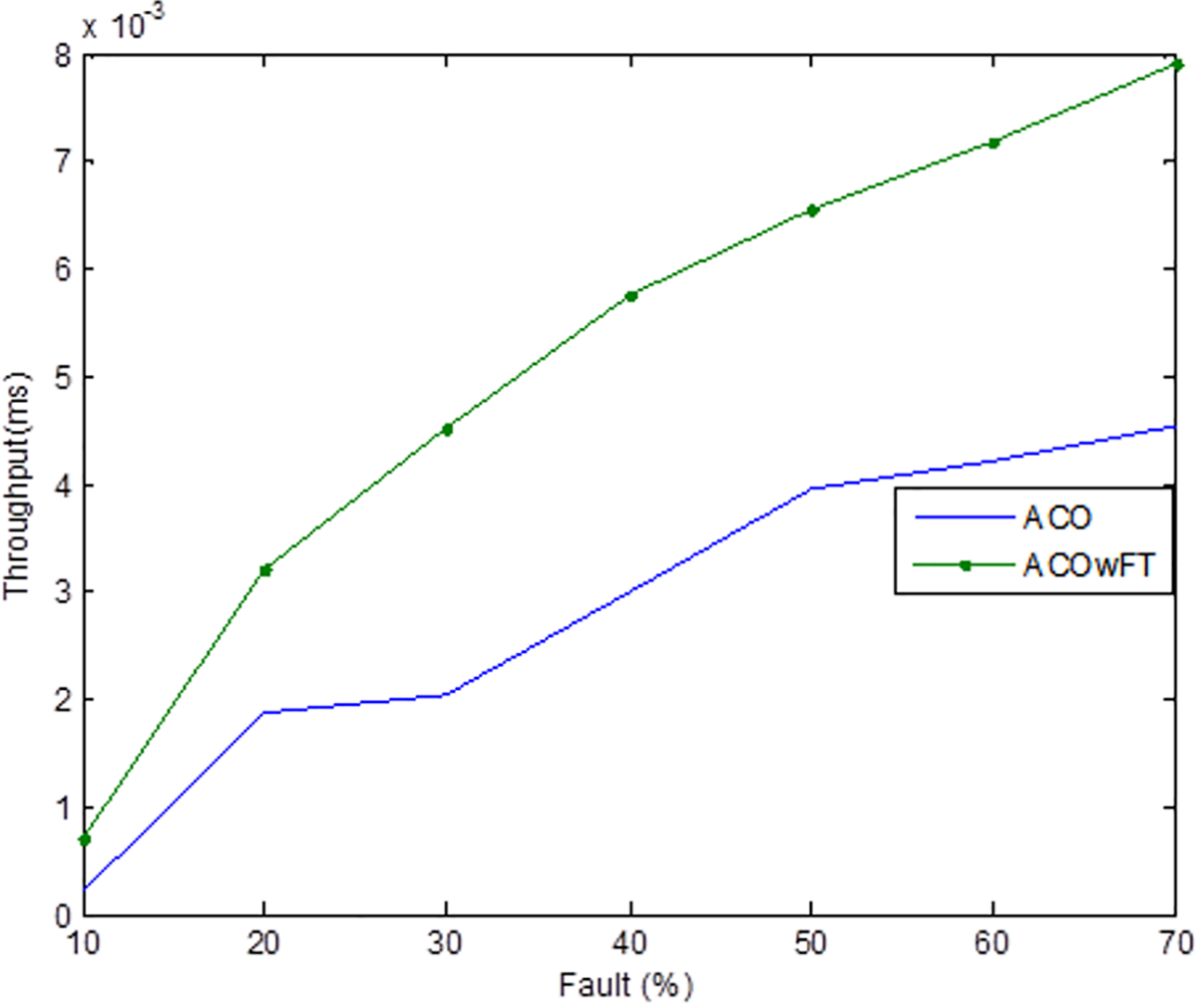
Average throughput time for varied number of faults (total number of Gridlets = 3,000).

**Fig 11:**
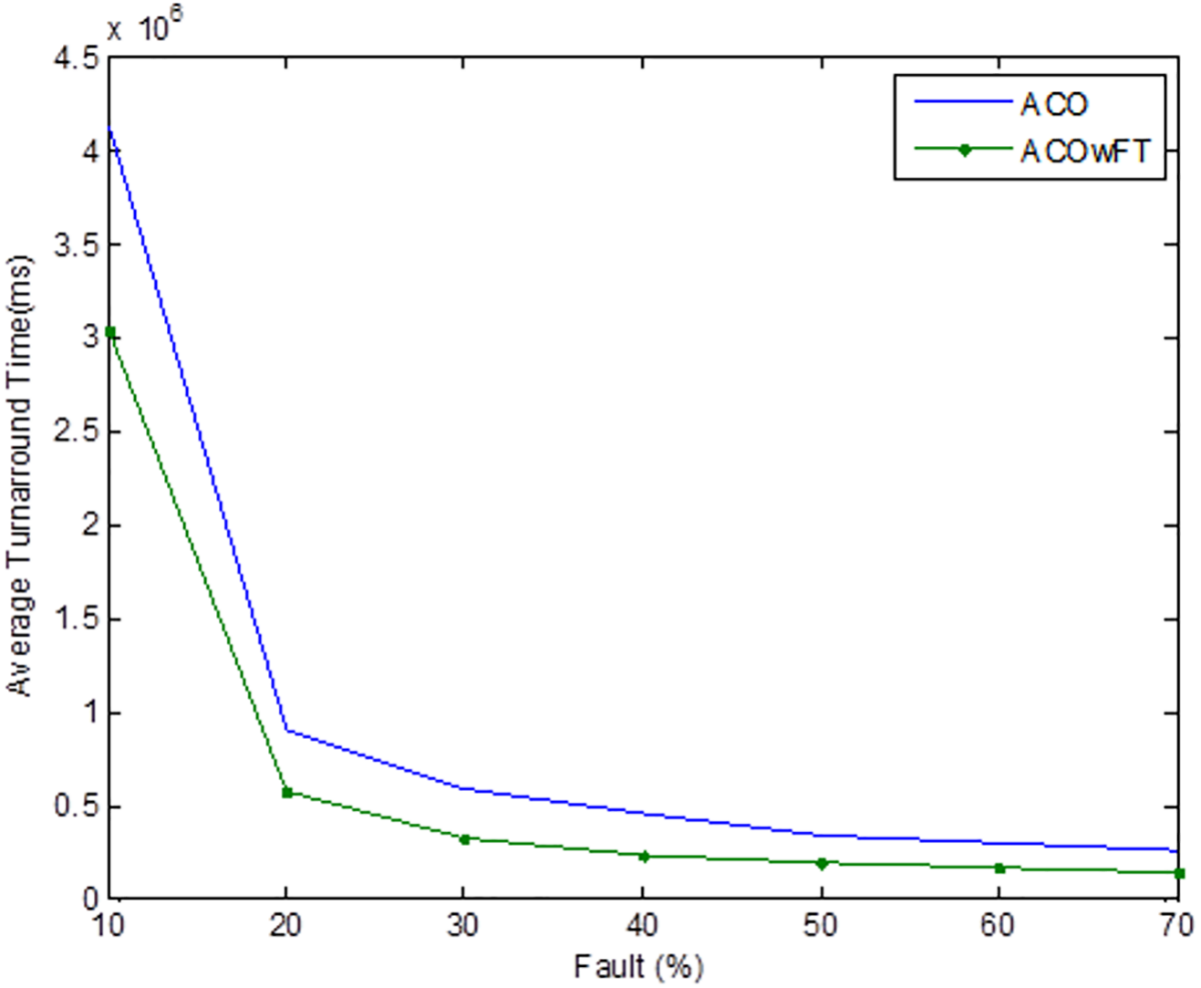
Average turnaround time for varied number of faults (total number of Gridlets = 3,000). To conclude the overall evaluation of the results, and with the aim of making a deeper analysis, the Friedman’s non-parametric test is carried out to check if there are any statistically significant difference between the two algorithms in terms of makespan, throughput, and turnaround time results reported for each of the algorithms. For makespan, throughput and average turnaround time, the resulting Friedman statistics has been 7.00. Taking into consideration that the confidence interval has been stated at the 99% confidence level, the critical point in χ^2^ distribution with 1 degree of freedom is 6.635. Since 7 > 6.635(*p*-value = 0.008), it can be concluded that there are statistically significant difference between the three metric results reported by ACOwFT and ACO whilst running χ^2^(1) = 7, with ACOwFT being the one with the lowest rank.

As can be seen in Figs 9, 10, and 11, the three metrics used for the evaluation of the two algorithms namely, makespan, throughput, and turnaround time are affected in the case of both algorithms as the percentage of faults introduced into the system increases. Generally, there were increases in both the makespan and average turnaround time, and a decrease in the throughputs. However, for the proposed ACOwFT algorithm, these changes are insignificant as compared to the results of the existing ACO algorithm. In the case of the throughput for example, the ACOwFT produced a better throughput than the ACO. This can be attributed to the fact that before any scheduling is done, the ACOwFT first considers the fault rate of the candidate resources to be allocated to the jobs and then makes it scheduling decision based on the same computed resources failure rates. This is not the case with the ACO, which does not have any fault handler mechanism. Similarly, the ACOwFT has a better turnaround time than the ACO, which can also be attributed to the fact that in ACO, there are more faulty resources compared to the ACOwFT, which has checkpointing mechanism and the capability to select resources with low failure tendency. Therefore, with more faulty resources in the ACO, it is expected that the delay time will likewise increase, as a result of the prolonged searching of alternative candidate resources, which leads to increase in the system turnaround time and the makespan as well.

## Conclusion

The paper describes the incorporation of a fault tolerance system, which is used to enhance the performance of the resource scheduling algorithm in Grid computing environment. This was achieved by introducing into the existing ACO a checkpointing mechanism. The ACO does not address the fault tolerance requirements explicitly. ACOwFT uses ACO algorithm, and builds fault tolerant solutions around it, to support checkpoint based fault tolerance. By adding fault tolerant features within the scheduling approach, the overall performance of the Grid system is improved.

The experimental results demonstrate that the proposed strategy effectively schedules the Grid jobs even in the presence of failures. It is observed from the experiments that the fault tolerance based resource scheduling strategy provides better results than the resource scheduling algorithm without fault tolerance in terms of various performance metrics, such as makespan, average turnaround time and the number of jobs completed. The proposed strategy has shown tangible improvements in satisfying the user’s QoS requirements for the submitted jobs in a fault tolerant way, in spite of the highly dynamic nature of the Grid.

The result of the two compared algorithms show that the proposed algorithm achieved up to 13% reduction in makespan, 12% maximization in terms of throughput and 12% maximization in average turnaround time when the Gridlets are varied and the resources are kept constant. Also when the Resources are varied and Gridlets are kept constant, the proposed algorithm achieved 18% reduction in makespan, 18% maximization in terms of throughput and up to 14% maximization in average turnaround time. The aim therefore, was to reduce the selection probability of resources with more fault occurrence history. Future work can consider some additional factors such as; jobs with a deadline, network delay, specifying QoS (Quality of Service) requirements and the consideration of checkpoint latency with respect to reading and writing to an external file. Another area of further research interest, which the new fault tolerance algorithm can be exploited, is the cloud computing environment. Similar experiments can be conducted to see how the algorithm performs in this setting by using the same performance evaluation metrics similar to the ones computed in this paper.
